# rTCT: Rodent Triangle Completion Task to Facilitate Reverse Translational Study of Path Integration

**DOI:** 10.1002/hipo.70090

**Published:** 2026-03-30

**Authors:** Stephen Duncan, Sulaiman Rehman, Vladislava Segen, Irene Choi, Sami Lawrence, Om Kalani, Lisette Gold, Lillian Goldman, Sophia Ramlo, Kylene Stickel, Dylan Layfield, Thomas Wolbers, Zoran Tiganj, Ehren L. Newman

**Affiliations:** ^1^ Department of Psychology & Brain Sciences Indiana University Bloomington Indiana USA; ^2^ German Center for Neurodegenerative Diseases (DZNE) Magdeburg Germany

## Abstract

Path integration is navigation in the absence of environmental landmarks and is a primary cognitive mechanism underlying spatial memory. Path integration performance is primarily assessed in humans using the Triangle Completion Task (TCT). In humans, TCT has shown promise for the early diagnosis of Alzheimer's disease. In rodents, however, path integration is assessed using a wide variety of tasks, but none of which currently provide a homologue for the TCT. As rodents are routinely used as preclinical models, homologous path integration tasks that result in comparable performance metrics between species are important. In the present study, we developed and tested a novel rodent version of the triangle completion task to enhance cross‐species comparability of path integration performance. Rats were able to comprehend and perform the task. A group of Alzheimer's disease model rats (TgF344‐AD) exhibited similar path integration performance to their wild‐type littermates. This work establishes a novel rodent homologue of the triangle completion task, facilitating enhanced reverse translational study of human path integration.

## 1 Introduction

Spatial memory enables animals to effectively navigate through their environment, playing a crucial role in their survival. One of the primary cognitive mechanisms underlying spatial memory is path integration, involving the use of self‐motion cues to navigate to goal locations in the absence of environmental landmarks (Etienne and Jeffery 2004). In humans path integration is most commonly assessed using the Triangle Completion Task (TCT; Segen et al. 2022) and has already shown promise for detecting Alzheimer's disease (AD) in its earlier stages (Howett et al. 2019; Newton et al. 2024; Segen et al. 2025). The regular use of TCT for assessing path integration in humans, and its potential for clinical application, make the TCT a valuable behavioral assessment tool in pre‐clinical research. In the Click or tap here to enter text. present study we developed and tested a novel rodent version of the TCT.

The TCT is a widely used test of path integration in humans (Allen et al. 2004; Harris and Wolbers 2012; Loomis et al. 1993; Mahmood et al. 2009; Marlinsky 1999; Nico et al. 2002; Stangl et al. 2020). During this task, participants are guided from location A to location B and then to location C before being asked to take the shortest path back to A without assistance from environmental markers. Participants must, therefore, use the idiothetic information gathered during the guided portions of the task to navigate back to the start location.

The utility of the TCT for assessing path integration performance to advance the mechanistic understanding of mental health has been clearly demonstrated. For example, Wiener et al. (2011) leveraged the TCT to dissociate cognitive mechanisms of human path integration. Harris and Wolbers (2012) identified significantly impaired path integration performance in elderly compared to younger human participants, using a virtual reality version of the TCT. The TCT also carries promise for advancing clinical diagnoses, with TCT ability in individuals with prodromal dementia differs significantly from health age matched controls (Howett et al. 2019; Newton et al. 2024; Segen et al. 2025).

The utility and clinical relevance of the TCT underscores the importance of having a homologous task in a rodent model. A rodent TCT would enable maximally relevant study of the neurobiological mechanisms of path integration, of path integration ability in transgenic models, and of preclinical assessment of therapeutic interventions. To retain the essential elements of the human TCT, a rodent homologue would allow for multiple trials a day consisting of a guided portion of the task where the animal is guided through the first two vertices of a triangular path before an unguided return, where the animal is rewarded for returning to the first location in the absence of environmental cues. There is no rodent homologue of the TCT currently in use.

Rather than TCT‐like tasks, rodent path integration is frequently assessed using a variety of tasks including path integration variants of classic tasks such as the Morris Water Maze and Barnes Maze (Barnes 1979; Benhamou 1997; Commins et al. 1999; Lee et al. 2023; Moghaddam and Bures 1996; Morris et al. 1982; Save and Moghaddam 1996; Whishaw and Gorny 1999) alongside more recently developed tasks (Bower et al. 2005; Guerrero et al. 2023; Najafian Jazi et al. 2023). While these tasks have proven utility for the study of rodent path integration; they differ from the human TCT in important ways. These include the availability of guiding cues during the return portion and/or use of threat escape as a means of motivating behavior rather than the purely appetitive motivations used in human studies. There have been some TCT‐like tasks described previously for use in several other species (Gorner 1958; Lindauer 1963; Müller and Wehner 1988; Seguinot et al. 1998) but those of primary interest here are those designed for rodents, as they are most frequently used pre‐clinical models of Alzheimer's disease (Alyan and Mcnaughton 1999; Seguinot et al. 1993; Whishaw and Gorny 1999). These tasks, however, still use methods not commonly found in human TCT tasks such as escape motivation and often involve a significant amount of experimenter interaction between trials and in guiding the animals to the target, leading to relatively few potential trials per day. There remains, therefore, a requirement for a homologous rodent version of the TCT which retains the essential elements of the human TCT.

Alzheimer's disease is a particularly relevant preclinical area of research and a rodent homologue of the TCT task would advance ongoing research. One of the first areas of the brain to suffer neurodegeneration is the entorhinal cortex (Jack and Holtzman 2013). The medial part of the entorhinal cortex has been shown to be instrumental in the ability of rats to perform accurate path integration (Gil et al. 2018; Jacob et al. 2017; Parron and Save 2004; Tennant et al. 2018; van Cauter et al. 2013). Spatial memory is one of the first cognitive functions to deteriorate in AD (Newton et al. 2024; Ritchie et al. 2018). Therefore, the development of sensitive and specific tests of spatial memory is potentially pivotal in the early diagnosis of the disease. Work in this area has already begun. Multiple studies have demonstrated that midlife deficits in the path integration performance of human participants, as measured by TCT, were predictive of AD risk (Howett et al. 2019; Mokrisova et al. 2016; Newton et al. 2024; Segen et al. 2025). Investigations into cognitive deterioration and its neural bases in early AD are necessarily split between human and rodent studies, with the promise that insights obtained in rodents will translate to human care. Yet, translational successes have been embarrassingly rare. The high failure rate is likely a product of the low external validity of rodent models (Pound and Ritskes‐Hoitinga 2018). This once again highlights the importance of unifying the assessment tools used across species.

To address the disparity in human and rodent path integration assessment, we developed and described a novel rodent version of the TCT used in humans that facilitates the comparison of path integration performance between humans and rodents—rTCT. We demonstrate that rats were able to perform the task, reliably completing multiple triangles per session. Furthermore, using a rat model of familial AD—TgF344‐AD (Cohen et al. 2013)—we evaluated the novel task's potential for studying AD‐associated spatial memory deficits.

## 2 Methods

The final goal of the present work was to develop a rodent homologue of the human triangle completion task—rTCT. The final task protocol was developed based on refinements made during the training of a primary cohort of rats and was tested/validated without further modification on a secondary cohort. The second cohort was trained only until age 6 months with the purpose of demonstrating that the final protocol was effective. The primary cohort was trained until age 13 months with the purpose of determining whether AD‐related transgenes resulted in overt task impairments.

We describe first the optimized version of the task which the secondary cohort performed that should be considered the final version. We then describe the protocol used to train the primary cohort of rats. Finally, we describe the data collection and analysis methods.

### 2.1 Subjects

All animal procedures were conducted in strict accordance with National Institutes of Health and the Indiana University Institutional Animal Care and Use Committee guidelines.

**FIGURE 1:**
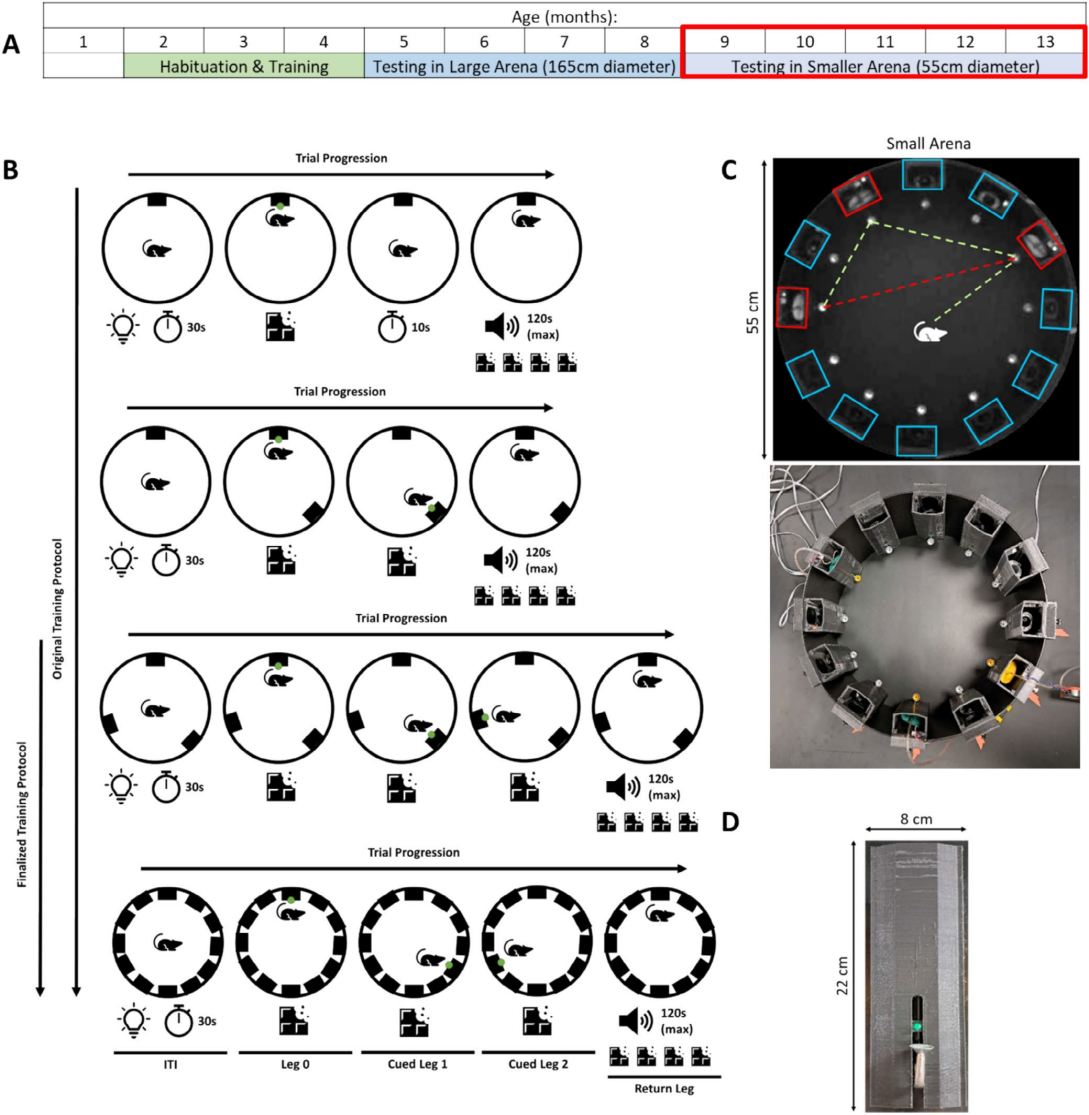
Triangle completion task apparatus and progression: (A) Timeline of the shaping and testing schedule that the animals underwent for the TCT. The data described here were gathered during the testing period highlighted by the red box. (B) Diagram representation of the stages of shaping used to steadily introduce the mechanics of the TCT to the animals. As shown the first two stages of shaping (rows 1 and 2) were removed in the finalized protocol. The different epochs of the task are labeled on the final row. These include the inter‐trial interval (ITI), all cued legs and the final return leg of a TCT trial. (C) The upper panel is a diagram demonstrating the full task where the rat runs between the three active dispensers (highlighted red) for the guided portion of the task (green dashed lines) before returning to the first dispenser visited (red dashed line). Decoy, non‐target dispensers are highlighted in blue. The lower panel is a photograph of the experimental set up for the smaller arena in which the testing took place. (D) A photograph of a liquid reward dispenser, showing the metal dispenser funnel at the front and the green LED above the funnel.

Thirty‐eight total rats were used in this study. Eighteen rats comprised the primary cohort. 11 (9F & 2M) of these animals were F344Tg‐AD, carrying mutant human amyloid precursor protein (APPsw) and presenilin 1 genes (PS1ΔE9; Cohen et al. 2013). 7 (1F and 6M) were age‐matched wildtype (WT) littermates. TgF344‐AD rats, developed by Cohen et al. (2013), exhibit AD‐associated, age‐dependent increases in amyloid beta and tau toxicity, along with various cognitive deficits. These animals started habituation at 2 months and finished testing at 13 months. Task performance from the primary cohort reported in this manuscript was obtained between months 9 and 13 (Figure 1A). Based on the performance of the primary cohort, the task shaping protocol was simplified and shortened. A secondary cohort was run to demonstrate the continued efficacy of the simplified finalized task protocol. The secondary cohort consisted of 20 rats. Ten carried the F344Tg‐AD transgenes (3F & 7M) and 10 were WT (3F & 7M). These animals were tested between the ages of 3 and 6 months.

Experimenters were blind to the genotype of each animal throughout behavioral testing and data scoring. Rats were bred in house. Original transgenic breeders were obtained from the Rat Resource & Research Centre (Columbia, MO) and wildtype breeders were purchased from Inotiv (Indianapolis, IN). Genotype was verified by Transnetyx using ear punches collected at postnatal day 21 during weaning.

Rats were either group or pair housed at all times and maintained on a 12 h light/dark cycle in a temperature and humidity‐controlled room with ad libitum access to water and food restricted to maintain ∼90% (85%–95%) of free feeding body weight. All work with the animals was performed during the light cycle. Daily handling began at P30.

### 2.2 Rodent TCT—Final Protocol

Rats completed a daily 15 min session in which they completed as many trials as possible. During a single TCT trial, rats are first guided between a series of three wall‐mounted liquid reward dispensers using LED cues. Then, cued by an audio tone, rats path integrate back to the first dispenser, in the absence of visual cues, to complete the characteristic triangular path of the TCT. This task structure mirrors that of the TCT regularly used to test human path integration. See Figure 1 for an overview.

#### 2.2.1 Apparatus

Behavioral testing was performed in a circular (55 cm diameter; 50 cm walls) arena surrounded by blackout curtains to occlude extra‐maze visual cues (Figure 1C). The diameter of the arena was chosen so that individual triangle leg lengths are approximately two rat body lengths, with the aim of aligning to leg‐lengths used in contemporary human TCT studies (e.g., Howett et al. 2019; Mokrisova et al. 2016; Stangl et al. 2020). Twelve dispensers were mounted to the walls of the arena at regular 30° intervals (Figure 1C). Though only three are needed to form the triangle on each trial, the remaining act as lures, probing for errors. We used 12 to balance the sensitivity of the task and the technical demand of constructing and controlling the apparatus. Individual dispensers consisted of plastic rectangular housing with an infra‐red (IR) LED mounted to the top pointing at the behavioral tracking camera mounted above the arena, a green LED facing the center of the enclosure and a metal reward delivery funnel mounted to the front (see Figure 1D). The top‐mounted IR LED was used to indicate the dispenser status to the behavioral tracking system as it was paired with the green LED so that they would turn on and off simultaneously. The green LED was selected to be bright enough to be reliably visible to the rat but not bright enough to illuminate the enclosure. The reward delivery funnel was selected to be aluminium to discourage rats from chewing the funnel between trials. The dispenser was hung such that the funnel top was about 12 cm from the floor, requiring rats to rear to check the funnel content and thereby allow us to distinguish between when a rat generates an overt response and when it passes by the dispenser. A custom system was built to allow for the remote liquid reward delivery to each of the dispensers. A computer‐controlled piston (a 100 mL syringe) was used to pump reward to each dispenser individually through a series of computer controlled solenoid valves via an Arduino interface. This system was controlled by the experimenter through a custom software interface. In addition to controlling reward delivery this software also allowed the experimenter control both the illumination of dispenser LED and visible illumination through a halogen lamp mounted above the arena. We refer to this light henceforth as the house light. Infra‐red (850 nm) LED strips are also suspended above the arena alongside an IR camera for monitoring and recording behavior irrespective of visible illumination. Additionally, the computer interface controlled a speaker, used to play a noisy audio tone (generated as white noise that was bandpass filtered to 2.7–3.3 kHz) for a prescribed duration. This custom reward delivery system and computer interface were built so that the experimenter and the animals need not interact during behavioral testing, reducing experimenter related behavioral confounds.

#### 2.2.2 Procedure

In complete darkness, rats were visually cued to approach and retrieve liquid reward from three dispensers, randomly selected from the 12 possible dispensers, before an audio cue signaled the availability of an increased reward at the first of the three dispensers. Consecutive illumination of the green LEDs on the front of each dispenser visually cued the rats to approach each dispenser in sequence. Once the rat retrieved liquid reward from the dispenser, the LED on the current dispenser was extinguished and the LED on the next dispenser in the sequence was illuminated. When the rat completed the guided portion of the task, retrieving liquid reward from each of the three dispensers, the return phase of the trial began as a noisy audio tone was played through a speaker near the data acquisition PC to indicate to the animal the presence of an increased (4×) reward at the dispenser visited first. Note that during this return phase, all dispenser‐mounted LEDs (green and IR) remained off so that no visual cues could support navigation to the target. The tone was played for a maximum of 2 min, during which the reward was available; after this, however, the trial was simply abandoned without reward. To promote rapid responses, the time for which the audio tone played was adjusted based on performance in the previous trial. If the previous trial was successfully completed within the time limit, the time limit for the current trial was reduced by 10 s. If the previous trial was unsuccessful, the current trial time limit was increased by 10 s. A lower limit was set to 15 s and an upper limit was set at 2 min. Trials were separated by a 30 s inter‐trial interval, during which the house lights were turned on. The following trial then took place using a different, pseudo‐randomly selected set of three dispensers. The only constraint on the random selection of dispensers was that no pair of dispensers could be immediate neighbors.

##### 2.2.2.1 Habituation

Before formal shaping began, rats were handled for 10–15 min daily by experimenters for 2 weeks prior to shaping. Rats then habituated to the reward dispensers in their home cages by hanging the dispenser from the wall of the cage. During this time, both the LED and audio tone were turned on in alternation and the dispenser baited to coincide with each. This served to train rats to associate the visual LED cue and the auditory tone cue with the availability of reward at the dispenser. Rats also habituated separately to the testing arena first in groups, along with their home cage mates, for 20 min and then individually for 10 min. This extensive habituation was performed in an attempt to manage the known anxiogenic phenotype of the TgF344‐AD animals (Pentkowski et al. 2018, 2022).

##### 2.2.2.2 Behavioral Shaping

Due to the relatively complex nature of the task mechanics, animal behavior was shaped in several phases prior to testing. This allowed the animals to learn the various task requirements and the sequence of events for a given trial. Across all phases, individual trials were run in a complete lack of visible light. As in testing, shaping sessions lasted 15 min, during which animals performed as many trials as possible. Upon reaching a criterion of three trials on two consecutive shaping days, animals progressed to the next phase. Preparing rats for the full task was performed as follows:

Stage 1: In the first stage of shaping, three dispensers were present on the walls of the arena and the animal was guided to and retrieved reward from each dispenser in sequence before the audio tone was played to signal the return phase where the rat attempted to return to the first dispenser for an increased reward (4×; Figure 1B, third row).

Stage 2: The second stage of shaping consisted of the exact same procedure as stage 1 but the positions of the dispensers were randomized at the start of each session. Dispenser positions were sampled from a total of 12 possible dispenser positions distributed uniformly at regular 30° intervals around the arena.

Full task: Finally, the full task followed the same procedure; however, all dispenser positions were occupied by dispensers (as seen in Figure 1B, fourth row) and the three dispensers forming the triangle were pseudo‐randomized between trials. The nine dispensers not active during a given trial served as decoys. All dispensers, active and decoy, contained liquid reward in their tubing throughout testing to ensure that active dispensers could not be distinguished by the scent of the liquid reward.

### 2.3 Primary Cohort Training Protocol Deviations

Here we describe the deviations from the final protocol described above that were used while training the primary rat cohort in which we compared aged task performance.

#### 2.3.1 Apparatus

Training and testing took place as described above with the following differences: We experimented with two other sizes of enclosure. The rats were run in a large (165 cm diameter) for ~4 months (mos. 5–8) and a small (45 cm diameter) enclosure for 1 week before we settled on the 55 cm diameter enclosure.

### 2.4 Procedure

Shaping of the animals to perform the task was done as previously described with the following minor differences:

Habituation: Rats were handled for 10–15 min daily by experimenters for 7 weeks prior to shaping (1.5–3 months). Habituation was extended while the experimental setup was refined. Rats were not trained to associate the audio tone and the liquid reward in the home cage during habituation to the dispenser.

#### 2.4.1 Shaping

Two additional stages of shaping were performed prior to the first stage described above (Figure 1B). These introduced the dispensers one at a time, starting with one dispenser in Stage A before introducing a second in Stage B before experiencing three dispensers at a time. However, these added complexity for little return in performance.

Stage A: A trial in the first additional stage of shaping consisted of the rat being guided to a single dispenser using the green LED with only a single dispenser present in the arena. Once the rat retrieved the reward, the LED was turned off. After a 10s pause, the unguided return phase of the trial began as signaled by the audio tone (Figure 1B, first row). The audio cue was played for a maximum of 2 min, during which the reward was available; note that the audio tone length was not dynamically adjusted during this phase.

Stage B: Two dispensers were present within the arena and rats were trained to visit each in the guided phase before returning to the first one in the return phase (Figure 1B, second row). Note that once again the audio tone length was not dynamically adjusted at this stage.

#### 2.4.2 Full Task

The full task proceeded as above; however, in this initial experiment the dispenser positions remained constant within a session meaning that the rats repeated the same triangle completion within a session. The triangles were, however, pseudorandomized between sessions. The ability to dynamically adjust the active dispensers between trials was not yet functional in our custom reward delivery system.

### 2.5 Behavioral Analysis

Animal behavior was recorded on an infra‐red camera suspended above the testing arena. Offline analyses using DeepLabCut (DLC) tracked the position of the nose, midback, and tail base (rump). Coordinates were imported into MATLAB for parsing and analysis.

#### 2.5.1 Markerless Pose Estimation

Rat tracking was performed offline with DLC. A new model was built using 20 frames from a video of each of the 18 rats from this study (360 total images) cropped to a tight circle surrounding the testing enclosure. The model was trained for about 400 k iterations before it was used to analyze the training videos and 20 “outlier frames” from each video were extracted based on the “jump” approach. After correcting the labels on the extracted outlier frames, the model was retrained with the 720 total frames for an additional ~1000 k iterations. DLC outputs were post‐processed in MATLAB using custom scripts to drop low confidence estimates and jumps. Missing data was replaced with linear interpolation.

#### 2.5.2 Video Annotation

Within each video, instances of specific features and events in task progression were annotated using the video recording and the DLC tracking data.

##### 2.5.2.1 Funnel Localization

The experimenter manually specified the position of the three active dispenser funnels using the Matlab function ginput.m. The remaining funnels were identified as peaks in luminance along the circumference of a circle drawn through these active dispenser locations. Each of the dispensers was then assigned a unique numerical label depending on its location in the arena.

##### 2.5.2.2 LED Localization

The experimenter also specified regions of interest around each of the infra‐red LEDs atop the active dispensers (IR‐ROIs).

#### 2.5.3 Dispenser LED and House Light Status

The video was processed frame‐by‐frame to extract the time‐varying mean luminance within the IR‐ROIs and across the whole video frame. Fluctuations in the whole frame luminance were thresholded to track when the overhead house light was off versus on, marking the inter‐trial intervals. Fluctuations in the IR‐ROIs were also thresholded to mark epochs when the LEDs were illuminated.

##### 2.5.3.1 Funnel “Pass‐Bys”

The behavior was parsed to identify the time of arrival and departure from each funnel. This was done by comparing the distance between the rat's nose and each of the dispensers for each frame of the video. A dispenser pass‐by occurred when the rat's nose passed within 25 pixels (5.5 cm) of a dispenser funnel and remained there for at least 0.5 s. Pass‐by events at the same dispenser separated by less than a second were considered the same event. A pass‐by could have included a nose‐poke or not.

##### 2.5.3.2 Funnel Nose‐Pokes

A nose‐poke is when a rat engages with a dispenser enough to occlude some of the funnel from the view of the overhead camera. Because this requires the rat to rear, it is used as an indicator of overt responding. Each frame within a cropped video of each dispenser funnel was compared to an image of the same funnel captured in a reference frame using MATLAB function immse.m. When the rats nose entered the funnel the luminance of the pixels within the frame changed relative to the reference frame allowing for the nose‐poke to be detected.

#### 2.5.4 Behavioral Epoch Definition

Behavioral epoching was done to mark the start and stop times of the key phase of each trial: Leg 0, as the rat approaches the starting point of the triangle (i.e., dispenser 1); Cued Leg 1, as the rat moves from dispenser 1 to dispenser 2; Cued Leg 2, as the rat moves from dispenser 2 to dispenser 3; and Uncued Return Leg, as the rat returns from dispenser 3 back to dispenser 1 (see Figure 1B final row). The start of Leg 0 was defined by the time that the LED on the first dispenser turned on. Leg 0 ended when the rat nose‐poked the first dispenser. The beginning of all remaining epochs were defined by the end of the pass‐by window of the previous dispenser, indicating that the rat left the immediate proximity of that dispenser. The end of each epoch was defined by a nose‐poke at the target dispenser. In the case of the return epoch—if the rat did not return during the audio‐tone‐indicated time limit the trial end was defined by the house light turning on (at the beginning of the inter‐trial‐interval) and the trial marked as incomplete.

#### 2.5.5 Quantification of TCT Performance

TCT performance was quantified using path length, latency, thigmotaxic index, tortuosity index, angular error, and incorrect dispenser visits.

*Path length* quantifies the distance traveled in a leg. Path length was calculated using the rump coordinate from DLC tracking, instead of the default nose coordinate, to prevent non‐locomotor head movements (e.g., grooming) from artificially extending the path length.

*Latency* is the duration of each leg, irrespective of how the time was spent.

*Thigmotaxic index* scored the tendency of the animal to remain against a wall during the leg. This was scored as:

$$I_{\text{thigmo}}=\left(t_{w}-t_{c}\right)/\left(t_{w}+t_{c}\right)$$

where $t_{c}$ and $t_{w}$ reflect the time spent in the enclosure center and along a wall, respectively. Positive values reflect more time spent near the walls versus in the enclosure center. The rat was scored to be along a wall if it was within ~4 cm of the wall. Note, in practice, this was achieved with a threshold of 12 cm from the apparent position of the upper rim of the enclosure as viewed from the top‐down camera.

*Tortuosity index* scored the ratio of the path length taken to the shortest possible path length. A value of one indicates the shortest possible path and larger values reflect how much longer the path length was than the minimum distance (e.g., 2 means twice the shortest path length).

*Angular error* was calculated as the angle between the direct path from the start to the target dispenser and the first principal component of the trajectory followed in the first 20 cm of the path after leaving the prior dispenser. Analysis derived from that used by Loomis et al. (1993).

*Incorrect dispenser visits* were defined by funnel nose‐poke visits to dispensers other than the target during a given epoch of the TCT trial.

#### 2.5.6 Confirmation of TCT Performance in the Second Cohort

A second cohort of rats was trained using the finalized training protocol with the purpose of confirming that the protocol effectively enabled rats to perform the TCT. To confirm that the protocol was effective, we examined whether rats were able to complete comparable numbers of trials per 15 min testing session and that their return latencies were at least as good as those observed in the primary cohort. This was achieved by analyzing the trial timing logs generated by the task control software. Because the second cohort was only trained until 6 months old and we did not expect meaningful path integration differences between the TG and WT rats at this age, no fine‐grained trajectory‐based analyses were performed.

### 2.6 Statistical Analysis

One‐sample *t*‐tests with Bonferroni corrections were used to assess if the proportions of epochs where correct and erroneous dispenser locations were visited was above chance levels. A two‐way mixed (Transgene × Arc) repeated measures ANOVA with Greenhouse–Geisser correction was used to assess the difference between errors on the short and long arcs relative to the target dispenser. Mann–Whitney *U* tests were used to assess differences in TCT performance between the transgenic and wild‐type animals, as measured by latency, path length, tortuosity index, thigmotaxic index, and angular error. Only epochs that were part of completed trials were used for these analyses. There were occasions on which a trial was terminated before it was finished as rats were given a maximum of 15 min to complete as many trials as possible.

## 3 Results

### 3.1 WT and TgF344‐AD Rats Are Able to Learn the Task Mechanics

**FIGURE 2:**
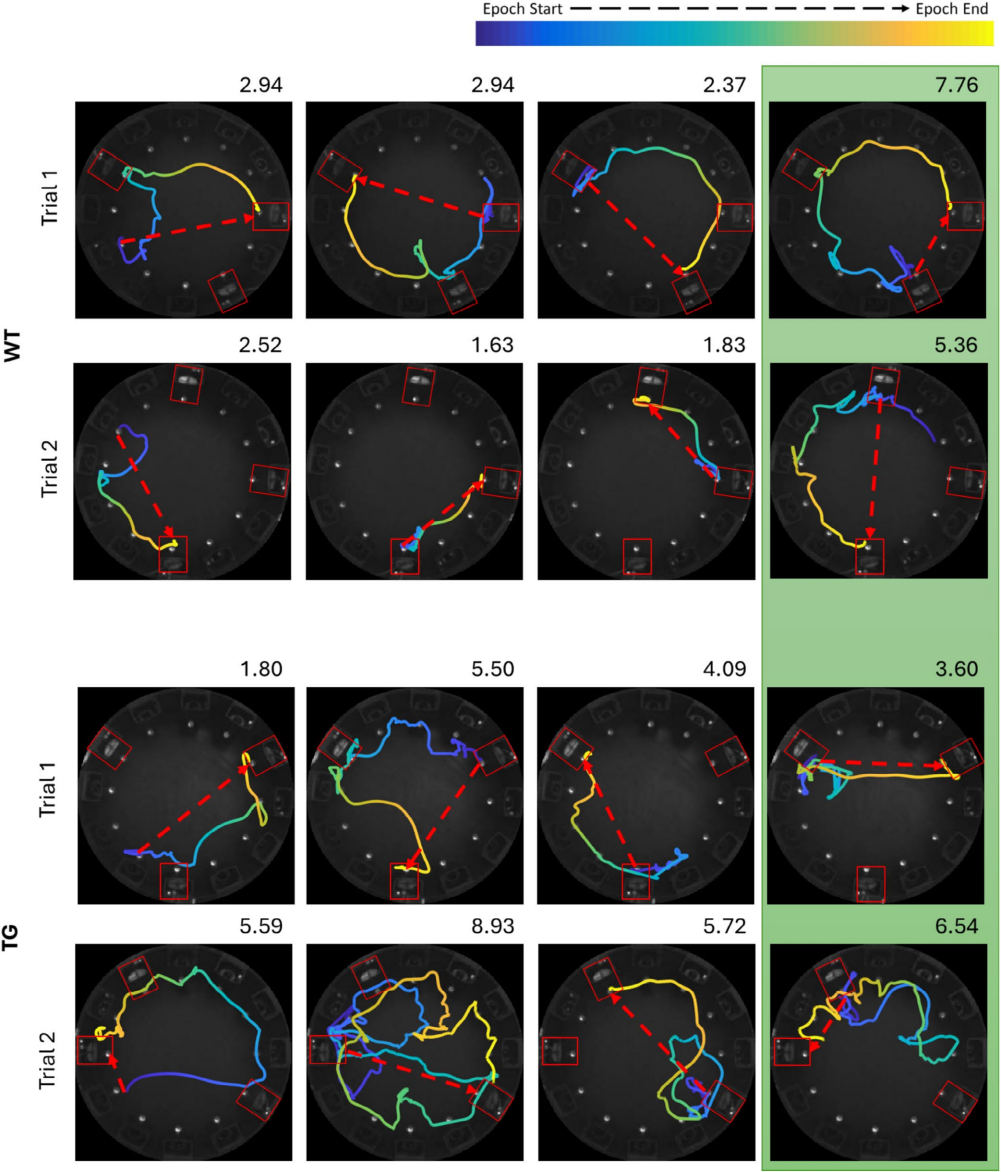
Representative rTCT trajectories: Example trajectories from two triangle completion task trials (top and bottom rows respectively) for both WT (top panel) and TgF344‐AD (bottom panel) animals. Columns 1–3 contain the 3 guided epochs and the 4th column (highlighted in green) contains the return epochs for each trial. The red dashed arrow indicates the shortest possible path for each epoch and the value at the upper‐right corner of each plot is the tortuosity index for that epoch.

Animals completed a total of 64 days of testing in the smaller arena (Figure 1A) resulting in 1100 videos. Tracking data was generated from videos recorded from an infra‐red camera suspended above the arena, using the behavior tracking software DeepLabCut. This allowed us to plot, parse, and analyze the animal's trajectories (Figure 2). Automated trajectory analyses were used to parse the behavior in each video—trajectories in 840 videos were successfully parsed and analyzed this way.

**FIGURE 3:**
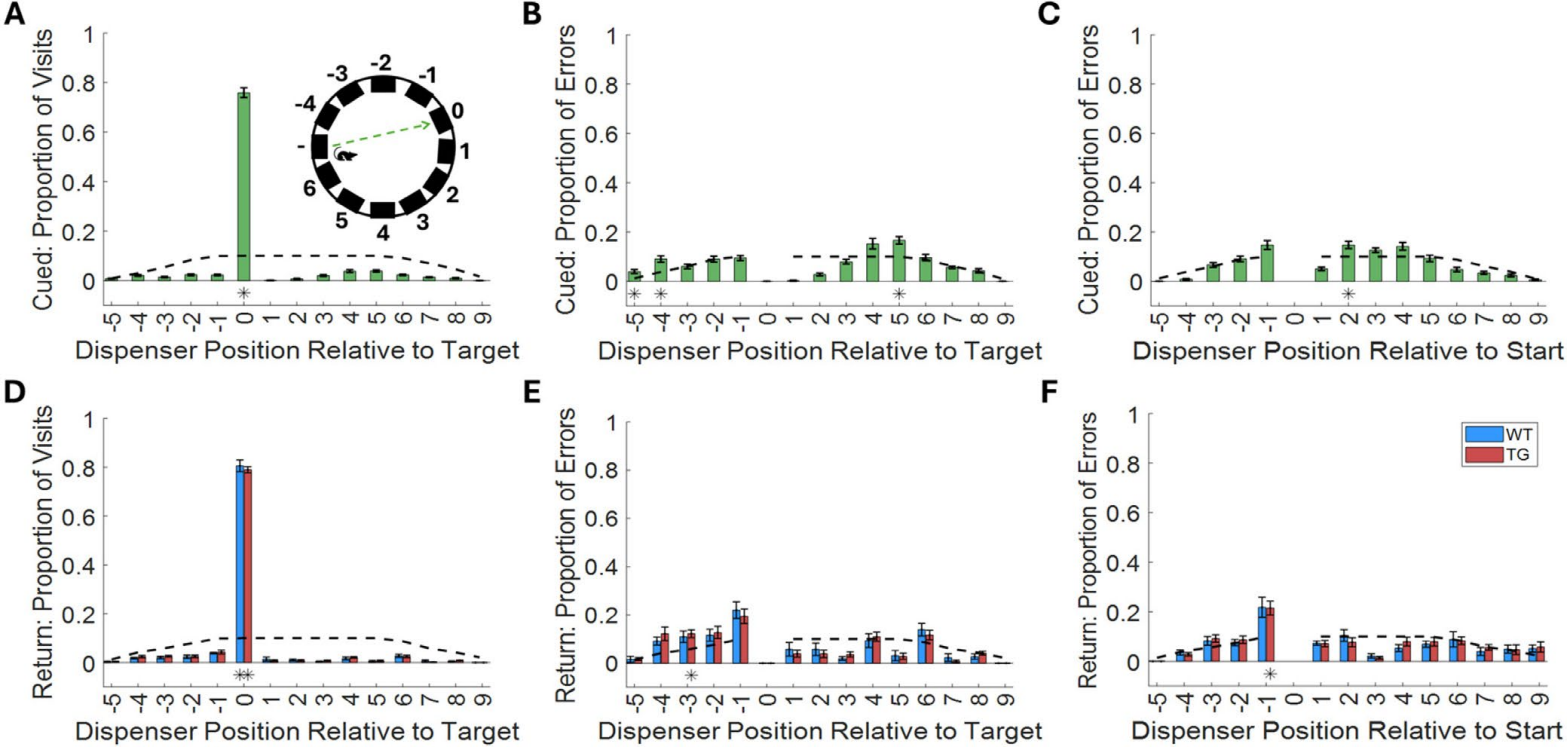
Rats comprehend rTCT mechanics: (A) Proportion of guided epoch visits made to each of the dispenser positions. Inset panel illustrates how dispensers were labeled: The target dispenser is labeled as 0 and non‐target dispensers are labeled by their position relative to the target with the shorter arc between the start and the target being labeled with negative integers. (B) Proportion of guided epochs with erroneous visits made to each of the non‐target dispenser positions. (C) Proportion of guided epochs with erroneous visits made to each of the non‐target dispenser positions. In this case the start dispenser is labeled as 0 and the remaining non‐target dispensers are labeled relative to the start instead of the target dispenser. (D) Proportion of visits made by WT (blue) and TgF344‐AD (TG; red) rats to each of the dispenser positions during the un‐guided return epochs. (E) Proportion of un‐guided epochs with erroneous visits made by WT and TgF344‐AD rats to each of the non‐target dispenser positions. (F) Proportion of un‐guided epochs with erroneous visits made to each of the non‐target dispenser positions. In this case the start dispenser is labeled as 0 and the remaining non‐target dispensers are labeled relative to the start instead of the target dispenser. (A–F) Black dashed lines indicate the chance level calculated for each dispenser position. A * below a bar indicates bars significantly greater than chance.

We first set out to establish whether rats were able to meaningfully comprehend the task mechanics and thus perform the rTCT. To examine their comprehension of the task mechanics in the guided epochs, we assessed the distribution of visits to each of the 12 available dispensers during these guided epochs. We labeled each of the non‐target dispensers using their position relative to the target dispenser (labeled as 0) and the starting location of that epoch (Figure 3A, diagram). Of the 12 possible dispensers, the animals exclusively visited the correct, target dispenser on 76% of guided epochs (Figure 3A). This performance is significantly greater than chance (*t*_(17)_ = 33.32, *p* < 0.001). This finding is consistent with the rats maintaining good comprehension of the guided epoch mechanics. Rats did not visit any other dispensers with a frequency above chance.

Focusing our analysis on the ~24% of guided epochs with errors (Figure 3B; excluding correct visits), we found that the dispenser at position 5 was visited with a frequency significantly above chance (*t*_(17)_ = 4.43, *p* < 0.001). As the dispenser in position 5 is directly opposite the target, it is possible that the errors at position 5 may be as a result of an erroneously flipped reference frame. Dispensers at positions −4 and −5 were also visited with a frequency significantly above chance (*t*_(17)_ = 4.18, *p* < 0.001; *t*_(17)_ = 3.63, *p* = 0.001, respectively). In this case it is possible that animals were frequently spot‐checking dispensers closest to the starting dispenser on their way to the target. To further examine this possibility, we next examined the positions of erroneously visited dispensers relative to the start dispenser instead of the target (Figure 3C). There was a trend towards animals visiting the dispensers directly adjacent to the start dispenser on the short arc suggesting they were spot‐checking. Only the dispenser at position 2, however, was visited on a significant proportion of error trials. Together, these findings indicate that the animals understood the task mechanics on cued legs; however, there was an indication of off task behavior.

### 3.2 WT and TgF344‐AD Animals Can Perform Path Integration in the rTCT

Following the three guided epochs of a trial was an un‐guided return epoch designed to assess path integration performance. This leg of the rTCT challenged the animals to navigate back to the first dispenser location in the absence of external landmarks (i.e., in complete darkness and without any LED indicator on the target dispenser), thus requiring them to path integrate (Figure 2, 4th column highlighted green). We first assessed the ability of animals to accurately complete the un‐guided epochs by measuring the proportion of visits to target vs. non‐target dispensers. We found that, on average, both TgF344‐AD and WT rats exclusively visited the target dispenser (i.e., without checking other dispensers *en‐*route) on ~80% of completed un‐guided return epochs (Figure 3D)—this performance was significantly greater than chance for both groups (WT: *t*_(6)_ = 29.50, *p* < 0.001; TG: *t*_(10)_ = 56.11, *p* < 0.001). The target dispenser was the only dispenser that rats visited significantly more often than chance when analyzed across all dispenser visits. When we restricted our analysis to return epochs containing errors (Figure 3E) we found that TgF344‐AD animals visited dispensers in position −3 on with a frequency significantly above chance (*t*_(6)_ = 4.44, *p* < 0.001). We also observed a trend towards increased visits by TgF344‐AD and WT rats to dispensers along the short arc to the target. Examining errors relative to the start dispenser revealed that TgF344‐AD animals visited the dispenser directly adjacent to the start dispenser on the short arc to the target significantly more often (*t*_(10)_ = 4.12, *p* < 0.001; Figure 3F). Overall, these results indicate that that the animals understood the objective of the task and were motivated to visit the target dispenser. When errors were made, they trended towards spot‐checks of dispensers along the shorter route to the target dispenser.

**FIGURE 4:**
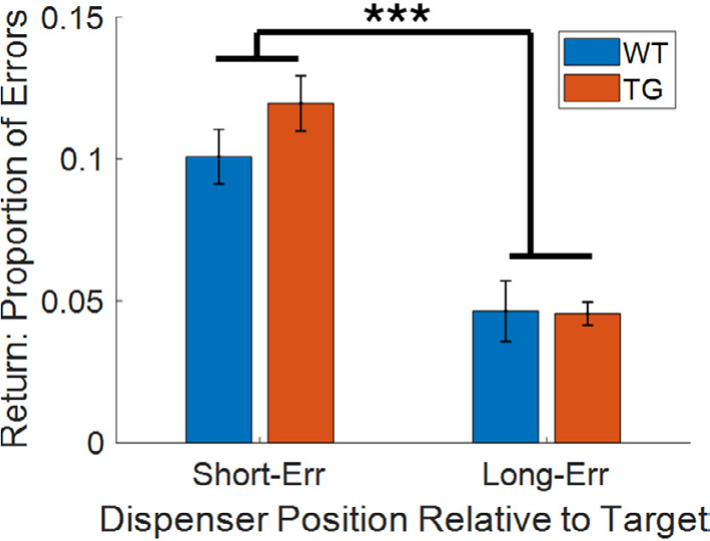
Animals made significantly more short errors than long errors on return epochs: Proportion of errors made by WT (blue) and TgF344‐AD (TG; red) rats to dispensers on the short‐arc (short‐err.) and long‐arc (long‐err.) between the start and target dispensers. ***p < 0.001.

The trend towards spot‐checking on the shorter arc was further investigated by comparing the total erroneous visits to the 4 dispensers adjacent to the target on the short versus long arcs (Figure 4; short‐err., long‐err., respectively). A two‐way mixed (Transgene × Arc) repeated measures ANOVA revealed that both TgF344‐AD and WT animals visited the short arc dispensers on a significantly greater proportion of errors (*F*_(1,16)_ = 62.04, *p* < 0.001). There was no transgene × arc interaction (*F*_(1,16)_ = 1.44, *p* = 0.25) or main effect of transgene (*F*_(1,16)_ = 0.93, *p* = 0.35). This result supports the conclusion that spot‐checking dispensers along the short‐arc between the start and target dispensers (i.e., a short stop error) was a primary source of errors during return epochs.

### 3.3 WT and TgF344‐AD Animals Exhibit Similar rTCT Performance

**FIGURE 5:**
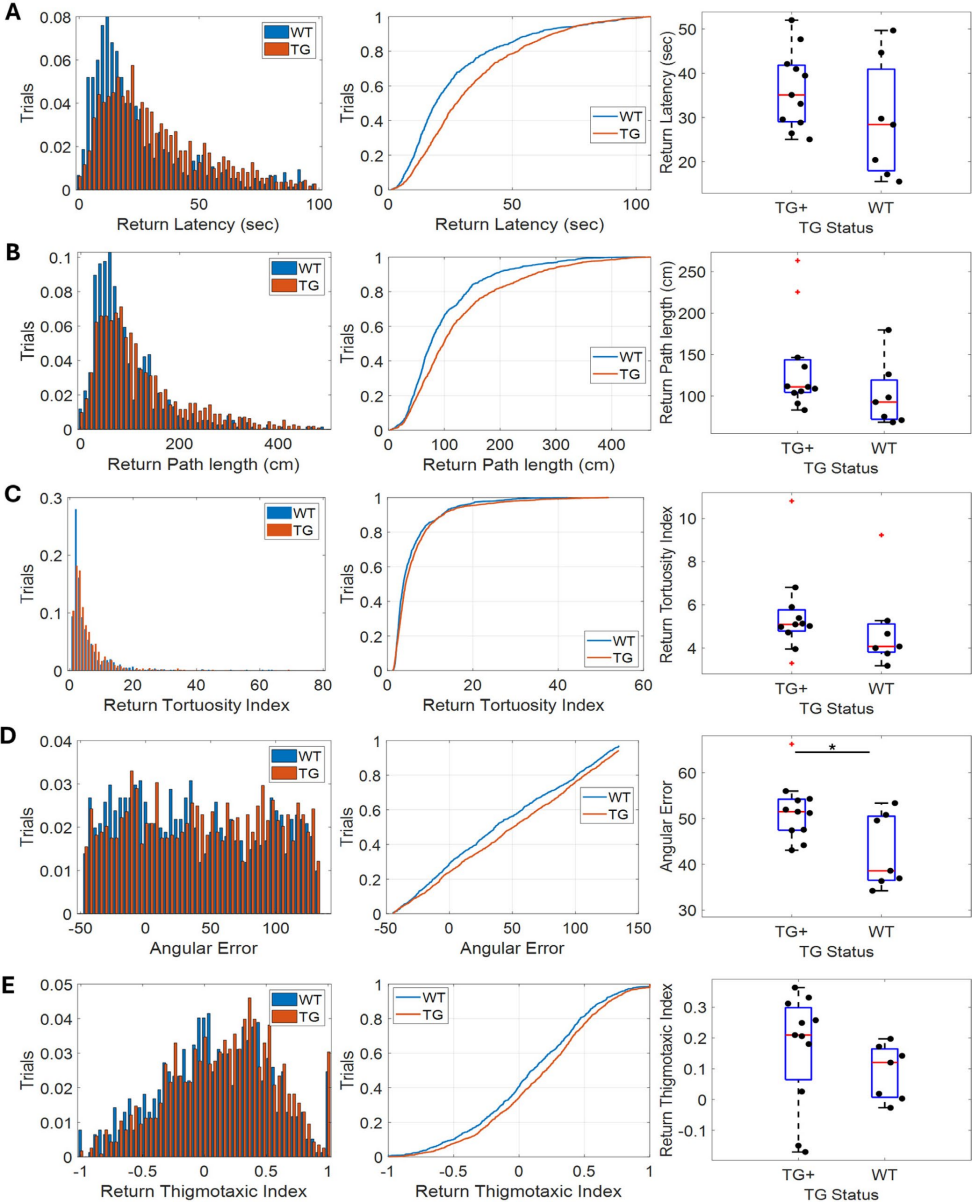
Both wild‐type and transgenic rats perform rTCT: Graphs showing (A) return latency, (B) path length, (C) tortuosity index, (D) angular error and (E) thigmotaxic index. The left hand column contains histograms; the middle column contains cumulative density plot and the right hand column contains box and whisker plots. These graphs all compare the performance of WT and TgF344‐AD animals during the return epoch of the TCT for each measure. ^+^Markers indicate outliers.

To test for potential fine‐grained differences between the transgenic TgF344‐AD rats and their transgene negative littermates, we examined the un‐guided return epochs using a variety of measures designed to capture path integration performance. These included return epoch latency, path length, tortuosity index, angular error, and thigmotaxic index (Figure 5). While we found that median angular error was significantly greater in the TgF344‐AD compared to WT rats (Figure 5D *U*_(16)_ = 15, *p* = 0.038); no significant differences were found in the median performance between the two groups on any of the other measures (TG+ vs. WT: Figure 5A, Latency—*U*_(16)_ = 25, *p* = 0.238; Figure 5B, Path Length—*U*_(16)_ = 20, *p* = 0.103; Figure 5C, Tortuosity Index—*U*_(16)_ = 24, *p* = 0.204; Figure 5E, Thigmotaxic Index—*U*_(16)_ = 19, *p* = 0.085). Together these results suggest TgF344‐AD and WT animals had largely equivalent performance. We noted, however, a consistent trend across measures wherein the performance of TgF344‐AD animals was marginally impaired compared to their WT counterparts, in addition to the TgF344‐AD animal's significantly greater angular error.

### 3.4 WT and TgF344‐AD rTCT Performance Is Unchanged When Examining the 1st Trial of Each Session Exclusively

**FIGURE 6:**
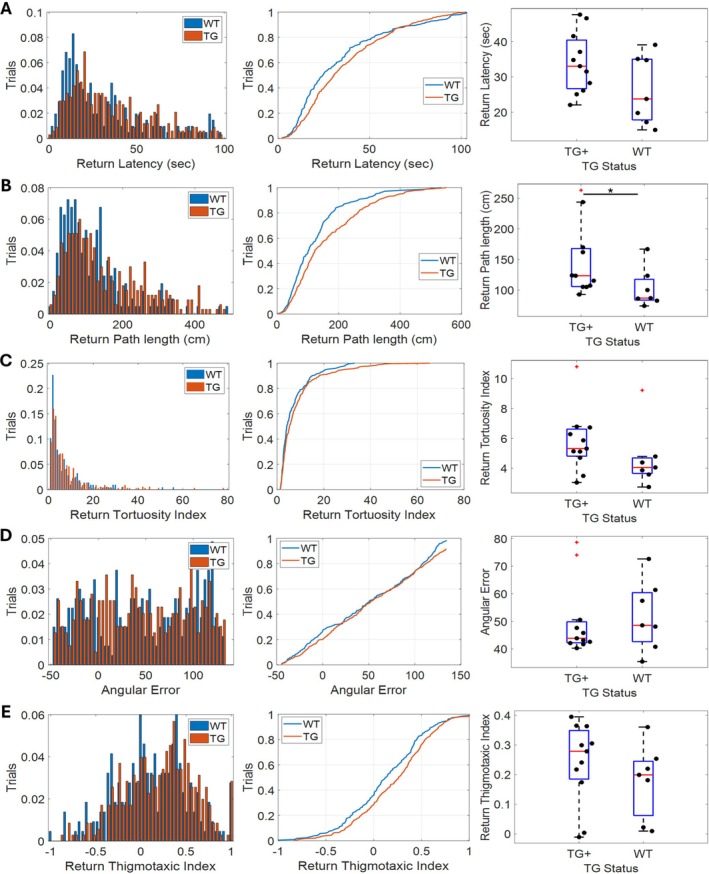
rTCT performance is unchanged if only using data from 1st trial of each session: Graphs showing (A) return latency, (B) path length, (C) tortuosity index, (D) angular error, and (E) thigmotaxic index. The left hand column contains histograms; the middle column contains cumulative density plots, and the right hand column contains box and whisker plots. These graphs all compare the performance of WT and TgF344‐AD animals during the 1st return epoch of each TCT session for each of the measures. *p < 0.05. ^+^Markers indicate outliers.

For this primary cohort of rats, the dispensers forming the triangle changed between sessions but remained consistent across trials within a single session. This raises the concern that, following the first trial, the requirement for path integration in this task was lost for the remainder of the session as rats could, in principle, rely on memory of prior trials in the session. To account for this, we next examined path integration performance using only the first trial from each session (Figure 6). In line with previous analyses, the performance was very similar between WT and TgF344‐AD animals with TG+ animals trending towards worse performance. Path length was statistically greater for TG+ rats compared to WT rats (*U*_(16)_ = 15, *p* = 0.038; Figure 6B) but the remaining measures were not significantly different between genotypes (TG+ vs. WT: Figure 6A, Latency—*U*_(16)_ = 22, *p* = 0.147; Figure 6C, Tortuosity Index—*U*_(16)_ = 21, *p* = 0.124; Figure 6D; Angular Error—*U*_(16)_ = 33, *p* = 0.653; Figure 6E, Thigmotaxic Index—*U*_(16)_ = 27, *p* = 0.317).

### 3.5 Guided Epochs Provide Control for Task Performance Independent of Path Integration Ability

**FIGURE 7:**
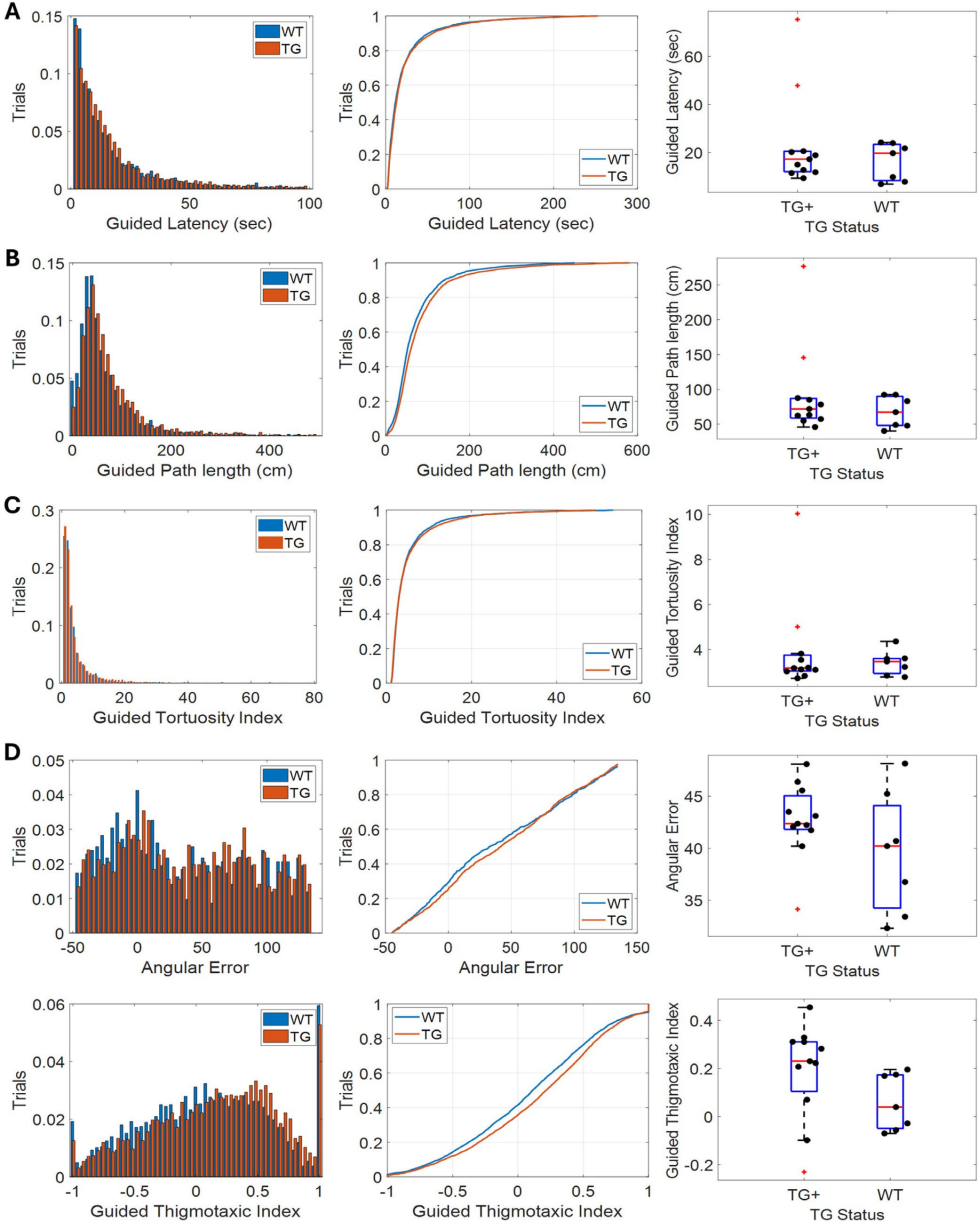
TgF344‐AD and WT animals exhibit similar performance on guided epochs: Graphs showing (A) return latency, (B) path length, (C) tortuosity index, (D) angular error, and (E) thigmotaxic index. The left hand column contains histograms; the middle column contains cumulative density plots, and the right hand column contains box and whisker plots.

It is important for a paradigm like rTCT to provide a means for identifying potential confounding factors between groups that may lead to apparent differences in path integration performance on return epochs. Guided epochs provide a means of identifying such factors as they have matched behavioral demands with the return epochs without the need to perform memory guided navigation. To that end we repeated the analyses described above for data collected during guided epochs to test for potential between group differences (Figure 7). Performance did not differ significantly between TgF344‐AD animals and WT counterparts indicating that both groups were well matched for basic task performance factors such as locomotor ability (TG+ vs. WT: Figure 7A, Latency—*U*_(16)_ = 35, *p* = 0.787; Figure 7B, Path Length—*U*_(16)_ = 32, *p* = 0.589; Figure 7C, Tortuosity Index—*U*_(16)_ = 35, *p* = 0.787; Figure 7D, Angular Error—*U*_(16)_ = 29, *p* = 0.412*;* Figure 7E, Thigmotaxic Index—*U*_(16)_ = 17, *p* = 0.057).

### 3.6 WT and TgF344‐AD Animals Exhibit Similar Kinematic Profiles During the Return Leg of rTCT

**FIGURE 8:**
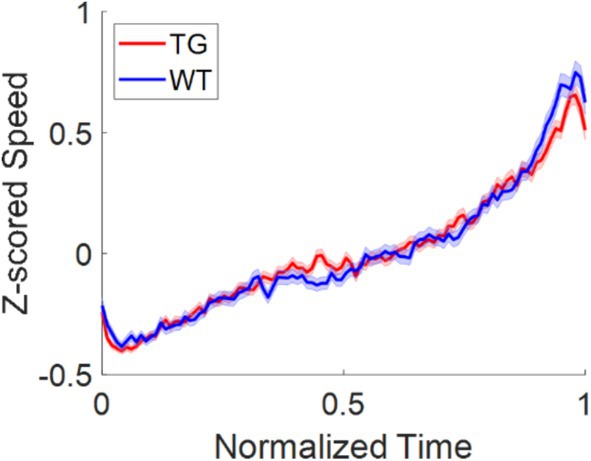
Both TgF344‐AD and WT animals reach peak locomotor speed at the end of their return trajectory: Locomotor speed graph comparing TgF344‐AD and WT animals, with normalized time and speed z‐scored over trials.

Because peak locomotor speed has previously been shown to differ between control and hippocampally lesioned animals (Wallace and Whishaw 2003), we additionally examined the kinematic profile of the TgF344‐AD and WT animals on the return epochs of the rTCT. However, we found no difference in the locomotor speed profile of the two groups, with both TgF344‐AD and WT animals reaching their peak running speed at the end of their trajectory (Figure 8).

### 3.7 Animals Trained Using the Finalized Training Protocol Exhibit Similar rTCT Performance

**FIGURE 9:**
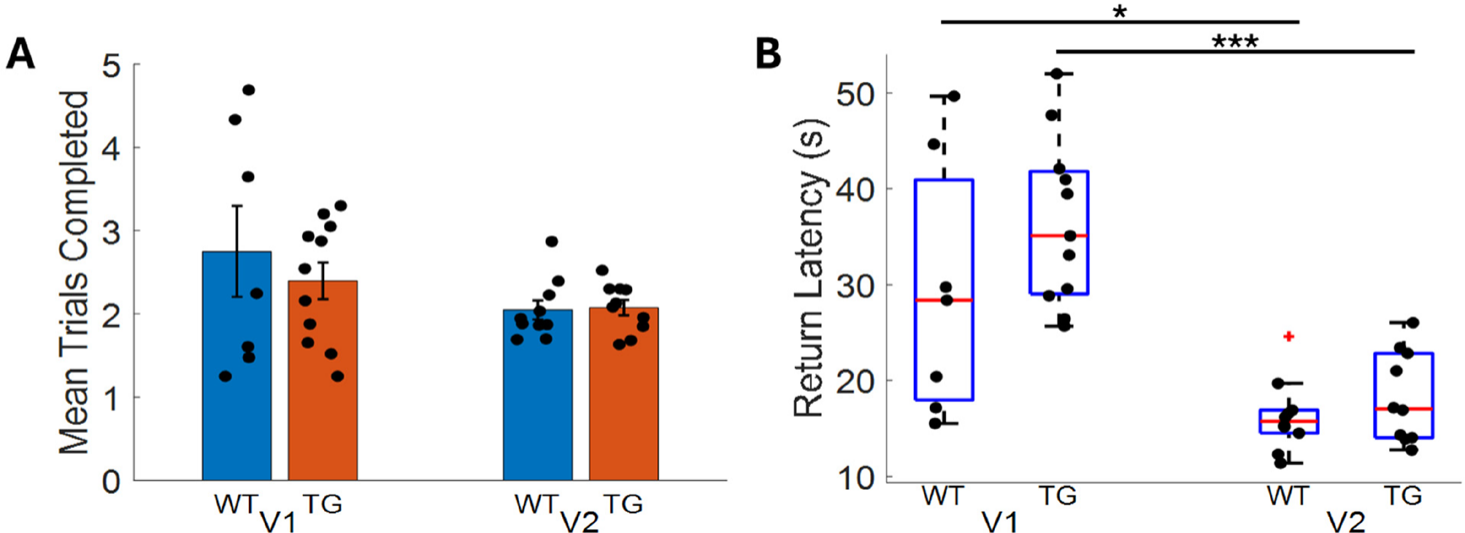
rTCT performance is maintained or improved through using the finalized training protocol: (A) Mean trials completed by the primary cohort who received the first version (V1) of the training protocol and by the second cohort who received the finalized (V2) version of the training protocol. (B) rTCT return latency observed following the first version (V1) of the training protocol and the finalized (V2) version of the protocol. *p < 0.05; ***p < 0.001.

A second cohort of rats was trained using the finalized training protocol, derived while training the primary cohort, to ensure that the final protocol effectively enabled rats to perform the rTCT. Following the initial training, this cohort completed 31 days of testing. Using these trials, we compared rTCT performance between the two groups. We found no significant difference in the number of trials completed per session between the two training protocols (Figure 9A). In the second cohort, the TgF344‐AD and WT both animals exhibited significantly faster return latencies (Figure 9B; WT: *U*_(15)_ = 8, *p* = 0.0027; TgF344AD: *U*_(19)_ = 1, *p* < 0.001), suggesting that rTCT performance after completing the finalized training protocol was as good or better than observed in the primary cohort, supporting the validity of the final protocol.

## 4 Discussion

Path integration has been studied extensively across both humans and rodents; however, the tasks used to assess it differ significantly between species. Homologous tests of path integration in humans and rodents are important as rodents are routinely used as preclinical models. In the present study, we developed a novel rodent version of the TCT, the rTCT, which retained the essential elements of the human variant of the task. In line with the human TCT, the rTCT included guidance through the first two legs of the triangle followed by and unguided return to the starting location in the absence of environmental cues (e.g., in darkness). Moreover, in contrast with previous rodent versions of the TCT, rTCT, like the human task, allows for multiple, uninterrupted triangle completions per session and thus the capacity for randomly varying geometries within a single session. Retaining these essential elements of the human task makes the rTCT ideal for the reverse‐translational study of cognitive decline in neurodegenerative diseases such as Alzheimer's disease. Rats were able to perform the rTCT, supporting the idea that this task could be used as a paradigm for preclinical work. Behavioral performance among TgF344‐AD rats and their WT littermates did not differ significantly at 13–16 months of age. We did, however, observe a consistent trend towards an impairment in the TgF344‐AD animals' performance. These results show that the rTCT, as a functional homologue to the human TCT, can facilitate reverse translational study of human path integration.

The rTCT differs from classic rodent path integration tasks in that it does not rely on escape as a motivation; like the human TCT, it is appetitively motivated. Examples of the classic tasks are path integration versions of the Morris Water Maze and Barnes Maze (Barnes 1979; Morris et al. 1982). The Morris Water Maze involves placing the animal into a circular pool filled with opaque water. The animal must then use path integration to find its way to a small platform, submerged just under the surface, in order to escape the water. In another task, the Barnes maze, the animal is placed on a circular platform featuring a number of evenly spaced holes around the perimeter. The animal must find its way to the hole which leads to an escape box. Both the Morris Water and Barnes mazes have proven utility in the assessment of path integration in rodents. Yet, their use of escape behaviors makes performance comparisons between these tasks and the human TCT challenging. The use of escape as motivation in these tasks may result in stress driven behavior, and the disruption of optimal spatial memory performance (Sandi and Pinelo‐Nava 2007; Schwabe and Wolf 2010). Differences in motivation can be consequential in reverse translational research. Distinct neural systems underly positively and negatively motivated behavior (Lammel et al. 2014; Namburi et al. 2015), potentially rendering conclusions performed with lab animals under one type of motivation irrelevant for human performance under another. A previous rodent homologue of the TCT developed by Whishaw and Gorny (1999) was based on the Barnes maze and shares some of these weaknesses as a result.

More recently developed spatial memory tasks bare a closer resemblance to the TCT (Bower et al. 2005; Guerrero et al. 2023; Najafian Jazi et al. 2023). In one task, dubbed AutoPI, a mouse searches a circular arena for a randomly placed lever which they operate in order to receive a reward upon returning to a home box (Najafian Jazi et al. 2023). Advanced apparatus and custom software are utilized to automatically progress each trial allowing for many trials per session. This impressive task is a useful test of path integration in rodents. The use of a home box, however, introduces an element of escape behavior as rodents prefer enclosed spaces to the open field of the testing arena. This differentiates this task from the TCT which uses appetitive motivation instead. Another spatial memory task which bares resemblance to the TCT was originally described by Bower et al. (2005) and more recently refined by Guerrero et al. (2023). Rats remain in an open field throughout testing and are guided between different locations around the perimeter of a circular platform using LED cues. Rats then return to one of two constant reward locations, depending on the sequence of locations visited during the trial, to receive a reward. This task has primarily been used to study sequential memory in hippocampus and has yet to be used to study path integration. Thus, the task setup and procedure require some modification to assess path integration in a manner similar to the TCT. For example, pseudo‐randomly changing reward locations in place of the two constant reward locations in the present version of the task. To effectively study path integration, the overlapping paths and fixed reward locations in the current task design may inadvertently introduce interference from olfactory cues. It is important to note that the differences between these tasks and the TCT do not call into question their validity or utility. These differences simply illustrate the remaining requirement for a further TCT homologue in rodents.

We showed here that 9–13 moth old TgF344‐AD rats broadly performed similarly well as their WT littermates in the rTCT. The lack of clear path integration performance differences in this study contrasts with the outcome one might expect from prior work. For example, TgF344‐AD rats show significantly impaired Morris Water Maze performance as early as 6 months of age (Bac et al. 2023; Berkowitz et al. 2018; Bernaud et al. 2022) and exhibit spatial memory deficits in an active place avoidance task (Chaudry et al. 2022). One reason might be due to differences in the motivation used to drive behavior. As noted above, behavior in these tasks, however, is motivated by escape. Thus, it is possible that previously observed impairments in TgF344‐AD rat performance could have been exaggerated by transgene related differences in escape motivation. The use of non‐appetitive motivation differs substantially from the positive reinforcers used in human path integration assessments and from what we used in our variant of the TCT. While TgF344‐AD rats have also been shown to have impaired performance in an appetitively motivated T‐maze based spatial working memory task (Saré et al. 2020) this task does not assess path integration ability. Thus, the behavioral deficits observed to date in TgF344‐AD rats may not be strongly related to human AD patient path integration ability. This further underscores the importance of establishing a rodent homologue for the human path integration assessment tools as we have done here.

The current results should not be interpreted to mean that TgF344‐AD rats cannot or will not model changes in human path integration ability. It is possible that the similar performance of TgF344‐AD and WT animals could be due to the state of neurodegeneration in associated brain areas in the 9–13 month age window. While tau hyperphosphorylation and neuronal loss in the hippocampus and entorhinal cortex have been reported in TgF344‐AD animals within the age range of the animals tested in this study, gross structural changes were observed later (Fowler et al. 2022; Morrone et al. 2022).

Path integration deficits found in human AD patients using the TCT (Howett et al. 2019; Mokrisova et al. 2016; Newton et al. 2024) were not all replicated in the present study. Path integration deficits have been observed in humans exhibiting mild cognitive impairment (MCI) as well as those with the APOE4 allele. The path integration deficits we observed in TgF344‐AD animals in the present study, however, were minimal. This disparity may be explained by several differences between the present study and those studying preclinical AD patients. Howett et al. (2019) found that human amnesic MCI patients had significantly greater distance error compared to controls. The task in the present study, however, is not sensitive to exclusive distance errors as all dispensers were mounted to the perimeter of the arena. This may be remedied in future by the addition of dispensers to locations at different distances from the arena perimeter. Newton et al. (2024) assessed human APOE4 allele carriers, the allele associated with the greatest risk of developing sporadic AD, and found that they APOE4 exhibited an average angular error of less than 10 degrees. This is reflected in the limited evidence of greater angular errors made by TgF344‐AD animals in the present study. It is possible that the 30 degree spaced dispensers in the present study did not provide the angular resolution to reliably detect angular errors of less than 10 degrees, as observed by Newton et al. (2024). This may be remedied in future by the addition of more dispensers.

The benefit of similar performance of the TgF344‐AD and WT rats, however, is that it enables better controlled comparison of the genotypes with respect to other dimensions. The lack of gross differences between the groups leaves it possible to compare neurophysiological differences between the genotypes in future work. For example, grid cells are hypothesized to support path integration (Gil et al. 2018) but lose tuning in rodent AD models (Jun et al. 2020; Kunz et al. 2015; Newman et al. 2014; Ying et al. 2022). Studying this effect benefits from having matched behavioral performance as this eliminates the confound of gross behavioral differences obscuring subtle neurophysiological changes in measures such as spatial tuning of neurons.

As this task is intended to assess pure path integration, it is important to make sure the animals are not simply following allothetic cues. It is difficult, however, to fully eliminate the possibility of allothetic cues being used by animals in tasks such as the rTCT. Several steps were taken, however, to remove the influence of visual and olfactory cues from the task arena. Visual cues were removed by conducting testing in darkness and in an arena surrounded by black ceiling‐height curtains. The experimenter can also, in principle, provide allothetic cues, such as olfactory or auditory cues. Thus, we ensured that the experimenter only interacted with the animal to place it in the arena and then remove it again at the end of the testing. Olfactory cues were reduced by thoroughly cleaning the apparatus between animals and making sure all dispensers contained liquid reward (even if they were never baited) to ensure active dispensers could not be distinguished from decoys through scent. Indeed, ideally olfactory cues would be removed through cleaning the apparatus between trials; however, this was not done in the present study to avoid excessive experimenter interaction. Future rodent versions of the TCT might include a lazy‐Susan mechanism to allow the further dissociation of allothetic and idiothetic cues (see Madhav et al. 2022; Najafian Jazi et al. 2023).

In the current study animals completed relatively few trials per 15 min testing session and often followed indirect trajectories between the start and target dispensers; making comparisons with the human version of the TCT more challenging. We attribute these limitations to off‐task behavior arising from lacking motivation in the current animals. We utilized food restriction to incentivize good performance; however, we found these animals, both TgF344‐AD and WT littermates, challenging to motivate. Our animals would become under‐conditioned before any improvement in motivation was observed. Tournier et al. (2021) observed similar trends in these animals, finding decreased motivation and lacking response to liquid reward. Liquid reward was the mechanism for reward delivery in the present study. A further possible explanation for low trial completion is the observed increase in anxiety levels in these animals, which pre‐dates cognitive decline (Pentkowski et al. 2018, 2022). While we took steps to reduce anxiety in our animals, it is possible that we were unsuccessful in entirely mitigating this factor. The fact remains, however, that despite the idiosyncrasies of this particular AD model, these rats were able to comprehend and perform the TCT. It follows, therefore, that with more easily motivated animals, the capacity of this task to detect specific differences in path integration performance may be increased. This highlights the need for an AD rat model better suited to cognitive behavioral testing, without the motivational and anxiety‐related features of the TgF344‐AD model.

In conclusion, the present study demonstrated that rats are able to perform a homologous path integration task to that performed in humans; facilitating the future comparison of human and rodent path integration performance. This enhanced intra‐species comparability makes this rodent TCT suitable for the reverse‐translational study of the deteriorating neurophysiology underlying path integration deficits in AD.

## Funding

This work was supported by the National Institutes of Health (RO1AG076198 to E.N.), Hutton Honors College research grants (to S.R., I.C., O.K., L.G.), Indiana University Office for Vice President for Research bridge funding, and Indiana University Office of Research integrated freshmen learning experience award (S.R. and S.R.). This research was supported in part by Lilly Endowment Inc., through its support for the Indiana University Pervasive Technology Institute and in part by Shared University Research grants from IBM Inc. to Indiana University.

## Ethics Statement

All animal procedures were conducted in strict accordance with National Institutes of Health and the Indiana University Institutional Animal Care and Use Committee guidelines.

## Conflicts of Interest

The authors declare no conflicts of interest.

## Data Availability

The data that support the findings of this study are available from the corresponding author upon reasonable request.
